# Gamma Irradiation of in-Shell and Blanched Peanuts Protects against Mycotoxic Fungi and Retains Their Nutraceutical Components during Long-Term Storage

**DOI:** 10.3390/ijms130910935

**Published:** 2012-08-31

**Authors:** Adriano Costa de Camargo, Thais Maria Ferreira de Souza Vieira, Marisa Aparecida Bismara Regitano-d’Arce, Severino Matias de Alencar, Maria Antonia Calori-Domingues, Marta Helena Fillet Spoto, Solange Guidolin Canniatti-Brazaca

**Affiliations:** Department of Agri-Food Industry, Food & Nutrition, “Luiz de Queiroz” College of Agriculture (ESALQ/USP), University of São Paulo, Av. Pádua Dias 11, P.O. Box 9, CEP, Piracicaba 13418-900, Brazil; E-Mails: tvieira@usp.br (T.M.F.S.V.); marisadarce@usp.br (M.A.B.R.A.); smalencar@usp.br(S.M.A.); macdomin@usp.br (M.A.C.-D.); martaspoto@usp.br (M.H.F.S.); sgcbraza@usp.br (S.G.C.-B.)

**Keywords:** gamma radiation, storage, mycotoxic fungi, color, water activity, nutraceuticals, polyunsaturated fatty acids, polyphenols, antioxidant properties

## Abstract

Peanut samples were irradiated (0.0, 5.2, 7.2 or 10.0 kGy), stored for a year (room temperature) and examined every three months. Mycotoxic fungi (MF) were detected in non-irradiated blanched peanuts. A dose of 5.2 kGy was found suitable to prevent MF growth in blanched samples. No MF was detected in in-shell peanuts, with or without irradiation. The colors of the control in-shell and blanched samples were, respectively, 44.72 and 60.21 (*L* *); 25.20 and 20.38 (Chroma); 53.05 and 86.46 (°Hue). The water activities (Aw) were 0.673 and 0.425. The corresponding fatty acids were 13.33% and 12.14% (C16:0), 44.94% and 44.92% (C18:1, ω9) and 37.10% and 37.63% (C18:2, ω6). The total phenolics (TP) were 4.62 and 2.52 mg GAE/g, with antioxidant activities (AA) of 16.97 and 10.36 μmol TEAC/g. Storage time negatively correlated with Aw (in-shell peanuts) or *L* *, linoleic acid, TP and AA (in-shell and blanched peanuts) but positively correlated with Aw (blanched peanuts), and with oleic acid (in-shell and blanched peanuts). Irradiation positively correlated with antioxidant activity (blanched peanuts). No correlation was found between irradiation and AA (in-shell samples) or fatty acids and TP (in-shell and blanched peanuts). Irradiation protected against MF and retained both the polyunsaturated fatty acids and polyphenols in the samples.

## 1. Introduction

Crude peanuts are of great importance in foods worldwide. Peanut food products are usually used by consumers from a wide range of socio-economic classes. However, according to Dorner [1] the contamination of peanuts with mycotoxins, particularly aflatoxins, is a worldwide problem that affects both food safety and agricultural economies. Most countries have adopted regulations that limit the quantity of aflatoxins in food and feed to 20 mg/kg or less; however, environmental conditions in most of the world where peanuts are produced and stored often make it difficult or impossible to attain such low concentrations. In addition, peanuts are known to be a source of allergenic proteins, a problem that manifests itself most often in children but also in adolescents and adults [2].

Irradiation of peanuts at 5.2 kGy [3] and 10.0 kGy [4] has been found to be an effective treatment to completely eliminate mycotoxic fungi in peanuts. According to Kilcast [5], ionizing radiation is, by definition, sufficiently high in energy to remove an electron from water, which is the main component of foods and living organisms, and to create highly reactive species, including free radicals such as the hydroxyl radical, and hydrogen peroxide. The authors also suggested that the predominant useful effects of irradiation rely on the reaction of these species with the DNA of microorganisms, causing death. In addition, Oh *et al*. [6] suggested that gamma radiation may reduce the allergenicity of peanut extracts. The authors reported that allergenic proteins that were exposed to irradiation presented distinct structural modifications as a result of aggregation, fragmentation, and the modification of amino acids, which, in turn, affected the solubility of proteins, their tertiary and secondary structure, and their immunogenicity. The alteration of epitopes by the denaturation of peanuts after irradiation may induce a lower response by T cells. The allergic reaction appears to be the result of a T_H_2-type T-cell response to one or more common environmental allergens [7].

Water activity is of enormous importance to fungi growth [8], oxidation and sensory changes caused by volatile flavor compounds [9], and in turn to the effects of gamma radiation. According to Bhushan, Bhat and Sharma [10], high water activity favors the decay of free radicals. Color is most likely the first issue regarding rejection raised by consumers. Several works have demonstrated that gamma radiation affects the color of nuts [11–13] and other food products submitted to this process in different ways.

The health benefits and disease-preventive effects of functional foods and nutraceuticals have primarily been concentrated in several areas. These include the treatment of cancer, atherosclerosis and other cardiovascular diseases, the process of aging, immune response-enhancing effect, as well as diabetes, among others [14]. Due to theirs high unsaturated and low saturated fatty acid contents, producers of peanuts and other nuts (*i.e.*, almonds, Brazil nuts, cashew nuts, hazelnuts, macadamia nuts, pecans, pine nuts, pistachio nuts, and walnuts) have received FDA authorization [15] to use the claim that “Scientific evidence suggests but does not prove that eating 1.5 ounces per day of most nuts as part of a diet low in saturated fat and cholesterol may reduce the risk of heart disease”. As mentioned before [5], irradiation causes molecular changes, among which the formation of free radicals is one of the most important for foods with a high fat content. Furthermore, the model proposed by Farmer *et al*. [16] shows that the formation of free radicals is the initial step in the mechanism of lipid autoxidation, which in turn can change the fatty acid composition of peanuts and consequently its functional benefits. Polyphenols such as proanthocyanidins [17] and flavonoids [18] have been isolated from the water-soluble fraction of peanut skins, demonstrating that the hydrophilic fraction of peanuts also provides benefits to the health of consumers. While there is a consensus on the oxidation effect of gamma radiation on the lipid fraction of food products, the same does not apply to the findings regarding the effects of this process on polyphenols and their antioxidant activity.

In previous work [19] regarding the oxidation stability of peanuts and tocopherol content, we provided evidence indicating that, if gamma radiation is considered an alternative for industrial-scale conservation, in-shell peanuts are the best feedstock. In the present study, in-shell and blanched peanut samples (cv. IAC-Runner 886) were subjected to doses of 0.0, 5.2, 7.2 or 10.0 kGy, stored for a year at room temperature and monitored every three months. To investigate the effects of irradiation and storage, determinations of mycotoxic fungi, water activity, color, fatty acid composition, total phenolic content and ABTS free radical scavenging activity were carried out.

## 2. Results and Discussion

### 2.1. Peanut Skin Color

The effects of gamma radiation and storage on color are shown in Table 1. Peanut skin color varies from light brown to deep red, and most color pigments in plants, especially red, purple and blue, belong to the flavonoid class of anthocyanins, with other flavonoid compounds acting as co-pigments [20]. Color can be affected by gamma radiation [11–13]. In the present study no color change due to irradiation was found in in-shell and blanched samples at time zero. In-shell peanuts had their *L* * (lightness) reduced at5.2 kGy (twelfth month), and at 7.2 kGy and 10.0 kGy (ninth month). The same was observed in blanched samples at 7.2 kGy (third month). An increase in *L* * in blanched samples was observed at 10.0 kGy (third and sixth months). No gamma radiation effect was observed in Chroma of in-shell samples during the whole storage and in blanched samples an increase was observed only at 10.0 kGy at the sixth month. °Hue decreased in in-shell and blanched samples at 7.2 kGy and 10.0 kGy (twelfth month).

According to previous work [21], the color of IAC-Tatu ST peanut cultivar was not affected by the short-term effects of gamma radiation at doses of up to 15.0 kGy. However, IAC-Runner 886 cultivar was affected with respect to *L* * and Croma, which was indicated by moderate darkening of this cultivar. Mexis and Kontominas [11], reported no short-term effect of gamma radiation on the color of raw peanuts (up to 7 kGy), which is in good agreement with the present work.

The lower the *L* * value of a sample is, the darker the product becomes. In the present study, at the end of the storage time all samples, with or without irradiation, presented lower *L* * values than those at time zero. Also it is worth noting that control and gamma-irradiated at 5.2 kGy in-shell samples presented lower *L* * only at the twelfth month of storage while the same behavior was observed from the ninth and third months of storage in samples irradiated with 7.2 kGy and 10.0 kGy, respectively. In view of this it is possible to suggest that gamma irradiation accelerated the decrease in *L* * value in in-shell samples. A negative correlation was found between storage time and *L* * in in-shell (*r* = −0.8147, *p* < 0.01) and blanched samples (*r* = −0.4401, *p* < 0.05). The colors of in-shell samples were the most affected by storage time (Table 2). Few changes were observed in Chroma and °Hue during storage time, but no correlation was found between storage time and Chroma or °Hue. Although peanut shells could have played a role in protecting the kernel, the results from the present work suggest that the shells were not efficient in preventing the changes to the color of the skins.

The blanching process involves heating, through which means Maillard compounds can be generated. According to Davis *et al*. [22], Maillard compounds present antioxidant properties. Several works [23,24] demonstrate that phenolic and antioxidant compounds are involved in redox reactions leading to color changes. In this way, browning reactions due to the oxidation of the kernels can be retarded by the antioxidant properties of Maillard compounds in blanched peanuts, which could explain the lowest correlation between storage time and *L* * in blanched samples. Free radical production due to water radiolysis is produced by gamma radiation. Thus oxidation changes, which cause color changes, can be influenced by moisture content and water activity. The present study demonstrated that the water activity of the samples was not the same (Table 3). Also the moisture content of the control in-shell sample (5.93%) was higher than that of the blanched control samples (3.38%), which along with the presence of Maillard compounds is helpful in explaining the lowest correlation between storage time and *L* *.

The results of the present study agree with the findings of Golge and Ova [25], who reported that three months storage had no effect on the *L* * (up to 5.0 kGy) of pine nuts. In the present study, the *L* * values were the most affected by storage, and °Hue was the least affected. If time zero is taken into account, no color effect was observed due to irradiation (Table 1), which in fact agrees with the study of Mexis *et al*. [26] which reported no short-time effect on the *L* * value of gamma irradiated (up to 3.0 kGy) raw unpeeled almond kernels. However, during long-term storage, the *L* * values decreased leading to a gradual darkening of the almonds especially in irradiated samples. According to the authors this effect was noticed even for samples packaged under N_2_. In the present study few of the differences observed occurred immediately after the third month of storage. Although significant differences were detected by Tukey’s test (*p* < 0.05), no correlation was found between irradiation and *L* *, Chroma or °Hue (Table 2); thus, it is possible to suggest that the differences could be due to the storage effect.

### 2.2. Water Activity

The water activities of gamma-irradiated and stored peanuts are presented in Table 3. The water activity of in-shell peanuts was lower at 10.0 kGy (ninth and twelfth months). Higher water activity was observed at 7.2 kGy (third month). The highest water activity in blanched samples was observed at radiation doses of 5.2 kGy (time zero) and 7.2 kGy (sixth month), while the lowest was observed at doses of 5.2 kGy (time zero, sixth and ninth months), 7.2 kGy (time zero, third and ninth months) and 10.0 kGy (time zero, third and sixth months). The water activity was measured as a possible helpful parameter to explain fungal infection as well as changes in fatty acid composition due to an oxidation process. The difference in the initial water activity of the in-shell and blanched samples can be related to the humidity of the samples. The control sample of the in-shell and blanched samples presented moisture contents of 5.93% and 3.38%, respectively. According to Nakai *et al*. [8] the water activity can be influenced by the drying process. Although some changes were noticed in the water activity of the samples these changes could not be explained by the irradiation process and may have been due to industrial drying of the samples. Furthermore the blanched samples were treated by heat in order to remove the skins. As well as in the drying process, the uniformity of the temperature on the samples could have caused the differences in the blanched samples.

**Figure 1:**
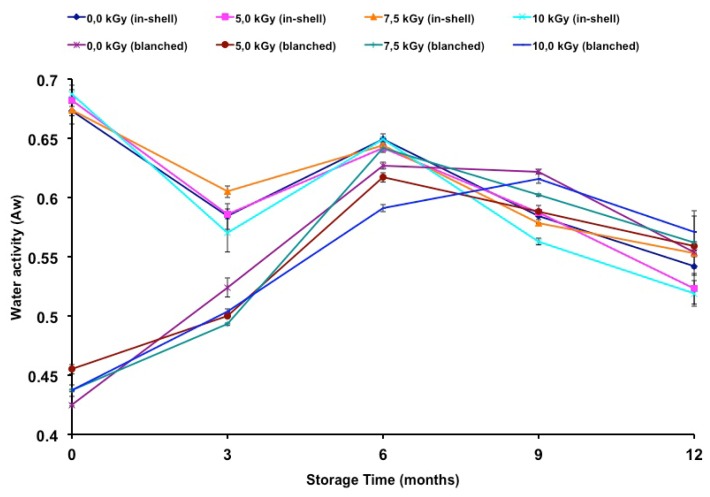
Water activity of gamma irradiated and in-shell and blanched peanuts during long-term storage. Error bars represent standard deviations of triplicate measurements.

Although peanut samples demonstrated significant differences according to Tukey’s multiple test (*p* < 0.05), no correlation was found between the gamma radiation and water activity of in-shell (*r* = −0.1890) or blanched samples (*r* = −0.0911). Figure 1 shows the change in water activity during storage time. Relative to t time zero, there was a decrease in the water activity of in-shell samples, gamma-irradiated or not. In contrast, an increase in water activity was observed in blanched samples, irradiated or not, throughout the entire storage period. A negative correlation was found between storage time and the water activity of in-shell samples (*r* = −0.7946; *p* < 0.01). A positive correlation was found between the storage time and the water activity of blanched samples (*r* = 0.7008; *p* < 0.01).

The storage time influenced the water activity of both in-shell and blanched peanuts. Nakai *et al*. [8] reported water activity that ranged from 0.38 to 0.62 (in-shell peanuts) and from 0.44 to 0.63 (peeled peanuts) for samples stored for twelve months. According to Reed *et al*. [9], oxidation and sensory changes in peanuts were higher at lower (0.19) than at higher water activity (0.60). In the present study, the in-shell samples presented higher water activity than those of the blanched samples until the third month of storage. Also it is worth noting that on the sixth, ninth and twelfth months of storage the water activities became similar between in-shell and blanched samples (Figure 1). Since water activity plays an important role in fungal growth as well as on the oxidation stability of food products it is correct to state that both kinds of samples became equally susceptible to both of these effects. In previous work [19], in-shell samples presented the lowest concentration of secondary oxidation compounds when compared to blanched and peeled samples. Thus, water activity could have played an important role in preventing the formation of secondary compounds, volatile compounds such as aldehydes and ketones, causing off flavors that are responsible for rejection by consumers. Water activity should be taken into account regarding oxidation stability and microbiological safety; however, other factors such as packaging, light exposure, the presence of antioxidants, moisture content, gas concentrations, pH, temperature and overall storage conditions should not be underestimated.

### 2.3. Mycotoxic Fungi

Table 4 presents the frequency of mycotoxic fungi in gamma-irradiated and stored peanuts. In-shell peanuts, irradiated or not, did not present mycotoxic fungi throughout the entire storage period. Positive results were obtained for non-irradiated blanched samples at time zero and at the third and sixth months of storage. Gamma-irradiated blanched peanuts (5.2–10.0 kGy) did not present potentially aflatoxigenic fungi. This result is in good agreement with the findings of Chiou *et al*. [3], whose studies demonstrated 5.2 kGy to be a suitable dose for fungi disinfestation in peanuts. In a previous study [19], we suggested that, regarding oxidation compounds and tocopherol content, in-shell gamma-irradiated peanuts were the best feedstock when compared to gamma-irradiated peeled and blanched samples. In the present study, in-shell samples were the best feedstock because no mycotoxic fungi were found during this kind of storage. Hilmy *et al*. [27] recontaminated gamma-irradiated (5.2 kGy) peanuts (a growth medium) to study the effects of atmospheric humidity on fungal growth and its toxin production by *Aspergillus flavus* after irradiation. According to the authors, although the lowest relative humidity of 91% (compared with 97%) and irradiation doses (0.5 kGy and 1 kGy) could not inhibit the aflatoxin B1 production of *Aspergillus flavus* in peanuts, the doses could delay the production by up to five and 14 days, respectively.

Gamma radiation cannot prevent recontamination; therefore, it is always necessary to protect gamma-irradiated products from recontamination sources. Insects are known to be vectors of mycotoxin-producing fungi. According to Nesci *et al*. [28], a strongly positive correlation was observed between peanut samples contaminated with insects and insects contaminated with *Aspergillus* section *Flavi.* Low doses of gamma radiation (0.2–0.8 kGy) are also efficient for killing and sterilizing insects (disinfestation of food) [29]. Nesci *et al*. [28] state that any action undertaken to reduce insect infestation during storage could help to reduce aflatoxin-producing fungi.

In addition to the recontamination issue, temperature and water activity play important roles in fungi growth. According to Horn [30], at temperatures of 15, 22, 30, 37 and 45 °C, *Aspergillus flavus* presented the highest growth at 22 °C–37 °C. In the present study, the storage temperature ranged from 23.06 °C to 25.01 °C (from time zero to the third month), 24.04 °C to 28.98 °C (from the fourth to the sixth month), 20.83 °C to 22.67 °C (from the seventh to the ninth month) and 20.95 °C to 23.35 °C (from the tenth to the twelfth month), temperatures that favored fungi growth. According to Nakai *et al*. [8], the growth of *Aspergillus flavus* was mainly influenced by temperature and relative humidity. The authors detected *Aspergillus flavus* growing at a water activity of 0.38 to 0.65, which is similar to the water activity observed in the present study (Table 3) but lower than that reported by Goncalez *et al*. [31], whose studies demonstrated higher *Aspergillus flavus* growth with water activity of 0.97.

As mentioned before [19], regarding tocopherol content and oxidation stability, gamma-irradiated in-shell samples were shown to be the best feedstock. Based on the data presented in Table 4, we also suggest that in-shell peanuts are less susceptible to mycotoxic fungi contamination. Furthermore, in the present study 5.2 kGy was demonstrated be the suitable dose to prevent mycotoxic fungi growth.

### 2.4. Fatty Acid Composition

Table 5 reports the fatty acid compositions of lipid extracts from gamma-irradiated and stored peanut samples. The oleic-to-linoleic acid (O/L) ratio is a quality index employed to determine the genetic characteristics of peanuts classified as normal, mid-, and high-oleic types, ranging from 1 to 1.5; 1.5 to 9.0, and above 9.0, respectively [32]. The present study was carried out with normal oleic peanuts. Palmitic acid, oleic acid, and linoleic acid were the major fatty acids. According to Andersen and Gorbet [33], the remaining fatty acids, stearic, arachidic, eicosenoic, behenic, and lignoceric acids, normally occur in weight percentages between 0.02% and 4.0%, which in fact agrees with values observed in the present study.

In a recent study, Shin *et al*. [32] analyzed 151 samples from two-year crops and noticed that there was a large variation with respect to the fatty acid content in samples classified as normal, mid-, or high-oleic. The authors reported that palmitic acid (C16:0) ranged from 5.31% to 11.49%; stearic acid (C18:0), 1.46% to 4.76%; oleic acid (C18:1, ω9), 44.78% to 82.17%; linoleic acid (C18:2, ω6), 2.85% to 33.92%; arachidic acid (C20:0), 0.87% to 2.18%; gondoic acid (C20:1, ω9), 1.09% to 3.13%; behenic acid (C22:0), 0.73% to 4.37%; and lignoceric acid (C24:0), 0.41% to 2.12%. These data are in agreement with those obtained in the current work.

Mexis and Kontominas [11,13] reported an increase in the saturated fatty acid content and a decrease in the mono- and polyunsaturated fatty acid content due to the irradiation of peanuts, pistachio and hazelnuts at doses of up to 7.0 kGy. However, according to another study by Mexis and Kontominas [12], monounsaturated fatty acids, as opposed to polysaturated fatty acids, were preferentially attacked by oxygen to produce primary and secondary oxidation products in gamma-irradiated cashew nuts.

Regarding the effects of gamma radiation on the major fatty acids, an increase in palmitic acid was observed at doses of 7.2 kGy (ninth month) in in-shell samples and 10.0 kGy (third month) in blanched samples (Table 5). The oleic acid content of in-shell samples was reduced by gamma radiation (7.2 kGy) at the third month of storage and increased at doses of 5.2, 7.2 or 10.0 kGy at the twelfth month. The oleic acid content of blanched samples increased at doses of 7.2 kGy or 10.0 kGy at the ninth month. The linoleic acid content of the in-shell samples decreased at the ninth month of storage (5.2 kGy) and at the sixth and ninth months of storage in blanched samples (7.2 kGy and 10.0 kGy). The saturated fatty acid content increased at the ninth month of storage in in-shell samples (5.2 kGy and 7.2 kGy) and at the third month of storage in blanched samples (10.0 kGy). The polyunsaturated fatty acid content decreased at the ninth month of storage in the in-shell samples (5.2 kGy) and at the sixth and ninth months in the blanched samples (7.2 kGy and 10.0 kGy). The monounsaturated fatty acid content decreased at time zero (10.0 kGy) and at the third month of storage (7.2 kGy) in in-shell samples and at time zero (5.2 kGy and 10.0 kGy) and at the third month of storage (7.2 kGy); the same decrease was observed in blanched samples. There was an increase at time zero (7.2 kGy) and at the sixth and ninth months of storage (7.2 kGy and 10.0 kGy) in blanched samples.

Regarding storage effects, a decrease in the palmitic acid content of in-shell samples was observed in samples submitted to 10.0 kGy (ninth month) of radiation. The oleic and monounsaturated fatty acid contents increased or remained unaffected during storage. Moreover, the linoleic and polyunsaturated fatty acid contents decreased or remained unaffected during storage. A decrease in the saturated fatty acid content of in-shell samples was observed at the ninth month of storage (0.0 kGy), and an increase was observed at the twelfth month (0.0 kGy) in blanched samples. The decrease in the polyunsaturated fatty acid content and the concomitant increase in the monounsaturated fatty acid content are explained by O’Keefe *et al*. [34]. The authors stated that the ratio of the oxidation rates of stearic, oleic, linoleic and linolenic acids was 1:10:100:200.

Table 6 shows Pearson’s correlation between gamma radiation and fatty acids as well as between storage time and fatty acids.

Only modest correlation existed between the stearic acid content of in-shell samples and gamma-radiation doses (*r* = 0.4935, *p* < 0.05). However, only a small concentration of stearic acid was found in the samples (Table 5). In turn, this correlation does not have a practical influence on the fatty acid profile. No correlation was found between gamma radiation and the remaining fatty acids of the samples. In-shell peanuts presented a positive correlation between storage time and oleic (*r* = 0.7239, *p* < 0.01), arachidic (*r* = 0.5927, *p* < 0.01), behenic (*r* = 0.6304, *p* < 0.01), lignoceric (*r* = 0.5760, *p* < 0.01) and monounsaturated fatty acids (0.7452, *p* < 0.01). The same was observed for oleic (*r* = 0.6181, *p* < 0.01), arachidic (*r* = 0.6167, *p* < 0.01), behenic (*r* = 0.6505, *p* < 0.01) and monounsaturated fatty acids (0.6397, *p* < 0.01) in blanched samples. A negative correlation was found between storage time and linoleic acids in in-shell (*r* = −0.7870, *p* < 0.01) and blanched (*r* = −0.7267, *p* < 0.01) samples. Linoleic acids are the only polyunsaturated fatty acids found in peanuts. In turn, a negative correlation was also found between storage time and polyunsaturated fatty acid content.

According to Finley and Shahidi [35], highly unsaturated fatty acids have been documented as having positive effects in reducing the risk of certain forms of cardiovascular disease, inflammatory diseases, as well as maternal and infant nutrition. If gamma radiation does not correlate negatively with the presence of polyunsaturated and monounsaturated fatty acids in peanuts, it can be inferred that there are no negative effects on the benefits offered to individuals who consume gamma-irradiated peanuts.

### 2.5. Total Phenolic Contents

The total phenolic contents of gamma-irradiated and stored peanuts are shown in Table 7. It has been noted in the literature that polyphenol contents vary between different peanut products. In gallic acid equivalents, peanut skins presented a total phenolic content ranging from 97.0 [36] to 143.6 [37], while peanuts, peanut butter [38] and peanut oil [39] presented contents of 3.96 mg/g, 5.36 mg/g, and 0.08 mg/g, respectively. The total phenolic contents of the non-irradiated in-shell samples are in good agreement with those reported by Wu *et al*. [38], while blanched samples presented lower concentrations than those in in-shell samples. The blanching process consists of removing the peanut skins; thus, as expected, the process changed the total phenolic content of the oilseed. Although peanut skins represent a small fraction of the kernel, on average 2.6% by weight [40], its removal represented a 45.4% loss in total phenolic content.

In the present study, few samples presented moderate differences due to irradiation. In-shell samples were negatively affected at 5.2 kGy and 10.0 kGy at the twelfth month of storage, while blanched samples were negatively affected at 5.2 kGy (third and twelfth months), 7.2 kGy (twelfth month) and 10.0 kGy (sixth month). A positive effect was also observed at 7.2 kGy and 10.0 kGy (third month) in blanched samples. In a previous study [21], no short-term effect was observed with respect to the total phenolic contents of peeled cv. IAC-Runner 886 peanut at radiation doses of up to 15.0 kGy. However, a positive effect was observed in cv. IAC-Tatu ST at doses of 7.2 kGy and 10.0 kGy. A random effect was reported by Toledo *et al*. [41] in soybean submitted to 2.0, 4.0 and 8.0 kGy of radiation. Štajner *et al*. [42] reported an increase in the total phenolic content of gamma-irradiated soybeans at doses up to 10.0 kGy. According to Harrison and Were [43], the increase in the amount of phenolic compounds due to gamma radiation could be attributed to their release from glycosidic components and the degradation of larger phenolic compounds into smaller ones by gamma radiation. Villavicencio *et al*. [44] reported no effect in gamma-irradiated Brazilian beans (var. Carioca and var. Macaçar) at doses of 0.5, 1.0, 2.5 and 5.0 kGy; however, a dose of 10.0 kGy decreased the total phenolic contents of var. Macaçar. Though Tukey’s test (*p* < 0.05) indicated significant differences due to irradiation, no correlation was found between irradiation and the total phenolic contents of in-shell (*r* = −0.0214) or blanched samples (*r* = 0.0326).

**Figure 2:**
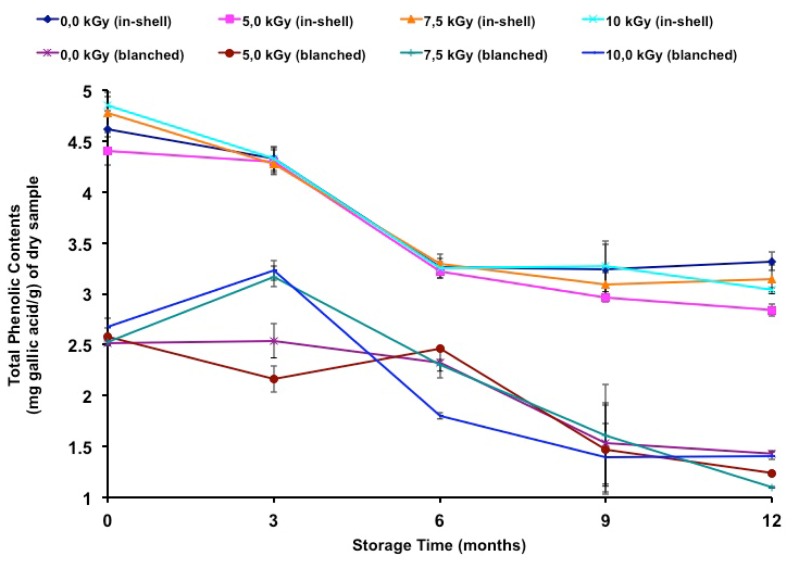
Total Phenolic Contents of gamma irradiated and in-shell and blanched peanuts during long-term storage. Error bars represent standard deviations of triplicate measurements.

In the present study, there was a decrease (Tukey’s test *p* < 0.05) in the total phenolic contents during the storage period (Table 7) and a negative correlation was found between storage time and the total phenolic contents of in-shell (*r* = −0.9101, *p* < 0.01) and blanched samples (*r* = −0.8591, *p* < 0.01). Figure 2 shows the total phenolic contents during long-term storage.

Nasar-Abbas *et al*. [45] reported that light exposure and temperature played an important role in the decrease in the total phenolic contents of stored faba beans. Additionally, Abramovič *et al*. [46] reported that there was a temperature effect on the polar phenolic content of stored Camelina sativa oil. Although some studies have reported gamma-radiation effects on the total phenolic contents of food products, there is still a lack of data that can explain the changes caused by gamma radiation. Thus, new studies should focus on this area of radiation chemistry.

### 2.6. ABTS Free Radical Scavenging Activity

Table 8 shows the results from an ABTS free radical scavenging activity assay. As mentioned before, the in-shell samples presented higher total phenolic contents than the blanched samples. Moreover, the ABTS free radical scavenging activity results of the in-shell samples were higher than those of the blanched samples. Removing peanut skin represented a 39.0% loss in ABTS free radical scavenging activity, which is in good agreement with the loss in the total phenolic content, which was approximately 45.4% (Table 7).

A decrease in the ABTS free radical scavenging activity was observed in in-shell samples at 5.2 kGy (sixth and twelfth months) and at 7.2 kGy (sixth month). In an earlier study [47], random effects due to gamma radiation (up to 10.0 kGy) were observed on the ABTS free radical scavenging activity of peanut skins. However, when ethanolic peanut skin extracts were applied in a model system of soybean oil (heated at 110 °C with an airflow of 9 L/h), the Oil Stability Index Rancimat method demonstrated that gamma radiation did not affect the antioxidant properties of the peanut skins. On the other hand, higher ABTS free radical scavenging activity was observed in gamma-irradiated blanched samples relative to that of non-irradiated samples at time zero (Table 8). At the third month of storage and later, no ABTS free radical scavenging activity was observed in blanched samples, irradiated or not.

Similar results were reported by Dixit *et al*. [48]. According to the authors, gamma-irradiated soybean genotypes showed an increase in antioxidant constituents and antioxidant properties at low doses of 0.5 kGy and 2.0 kGy, while the antioxidant effects of soy seeds either decreased or remained constant at a higher dose of 5.0 kGy. These findings are in good agreement with those of the present study with respect to the short-term effects (time zero) of gamma radiation on ABTS free radical scavenging activity. Although significant differences have been observed in in-shell and blanched samples (Tukey’s test, *p* < 0.05), no correlation was found between gamma radiation dose and ABTS free radical scavenging activity in in-shell samples (*r* = −0.1005). On the other hand, positive correlation was found between irradiation and the ABTS free radical scavenging activity of the blanched samples (*r* = 0.9355, *p* < 0.01).

There was a decrease in ABTS free radical scavenging activity during the storage time (Table 8). Only modest negative correlation was found between storage time and the ABTS free radical scavenging activity of in-shell samples (*r* = −0.5779, *p* < 0.01). Talcott *et al*. [49] reported a significant decrease in ORAC (oxygen radical absorbance capacity) in normal, mid-, and high-oleic acid runner peanut cultivars stored for four months at different temperatures (20 °C and 35 °C). According to the authors, free *p*-coumaric acid, three esterified derivatives of *p*-coumaric, and two esterified derivatives of hydroxybenzoic acid were the predominant polyphenolics present and their rates of change were similar among cultivars and independent of storage time or temperature. However, only modest correlations existed between total phenolics and antioxidant capacity. The authors stated that decreases during storage were a good indication that soluble (80% methanol) phytochemicals with antioxidant capacity, most likely polyphenolics and/or protein-polyphenolic complexes, had altered during storage. In the present work, though phenolic compounds were present in blanched samples during the whole storage (Table 7), the decrease in their concentration along with molecular alterations could have influenced their ABTS free radical scavenging activity.

Phenolic and polyphenolic compounds constitute an important class of secondary plant metabolites that act as free radical scavengers and inhibitors of LDL cholesterol oxidation and DNA breakage, among other roles they play. Indeed, the role of food phenolics and polyphenolics in the prevention of cardiovascular disease and certain types of cancer is well recognized [14].

As mentioned before, no correlation was found between gamma radiation and polyunsaturated or monounsaturated fatty acids. Furthermore, no negative correlation was found between gamma radiation and total phenolic contents or ABTS free radical scavenging activity. Thus, after one year of analysis, it can be concluded that irradiation protected against mycotoxic fungi growth (microbiological safety) and retained the nutraceutical components of the samples during long-term storage.

## 3. Experimental Section

### 3.1. Material

Samples of in-shell and blanched cv. IAC-Runner 886 (crop year 2009/2010) were obtained from CAP—Agroindustrial, Dumont, São Paulo State, Brazil.

### 3.2. Methods

#### 3.2.1. Irradiation Process

In-shell and blanched peanut samples (cv. IAC-Runner 886) were separated into 1.5-kg portions and placed in polyethylene plastic bags. The bags (excluding “controls”) were irradiated at doses of 5.2 ± 0.2; 7.2 ± 0.2, and 10.0 ± 0.35 kGy at a dose rate of 7.5 kGy/h. A Harwell Perspex polymethylmethacrylate Amber 3042 dosimeter (PMMA Instruments, Harwell, UK) was used to measure irradiation doses. The irradiation process was carried out in the city of São Paulo, São Paulo State, Brazil using a multipurpose Cobalt-60 γ-irradiation apparatus from the Nuclear Energy Research Institute (IPEN). IPEN is an autarchy associated with the University of São Paulo—supported and operated technically and administratively by the National Nuclear Energy Commission (CNEN). The samples were irradiated in air at room temperature (25 °C). The samples were stored for twelve months at room temperature, and analyses were performed at the beginning of the study and after three and six months of storage. The storage temperature was monitored by a thermo-hygrometer (RH520A, Extech Instruments, Nashua, NH, USA) and ranged from to 23.06 °C to 25.01 °C (from time zero to the third month), 24.04 °C to 28.98 °C (from the fourth to the sixth month), 20.83 °C to 22.67 °C (from the seventh to the ninth month) and 20.95 °C to 23.35 °C (from the tenth to the twelfth month).

#### 3.2.2. Color Measurements

*L* *, *a* * and *b* * values were measured using a HunterLab colorimeter (Colorflex Model 45/0, HunterLab Inc., Reston VA, USA) previously calibrated with a white reference standard tile. In-shell and blanched peanuts were evenly spread on a Petri dish and analyzed. *L* *, *a* *, and *b* * values correspond to lightness/darkness, redness/greenness, and yellowness/blueness, respectively. °Hue, an attribute of color perception, was calculated from *a* * and *b* * values using the following formula: °hue angle = [arc tan (*b* */*a* *)]. Chroma was calculated using the following formula: Chroma = (*a*^2^ + *b*^2^)^1/2^.

#### 3.2.3. Water Activity

Water activity (*aw*) was determined using an Aqualab CX-2 apparatus (Decagon Devices, Inc., Pullman, WA, USA).

#### 3.2.4. Mycotoxic Fungi

In-shell and blanched peanuts were analyzed by direct plating onto *Aspergillus flavus-parasiticus* Agar (AFPA) according to the described by Pitt *et al*. [50]. In-shell peanuts were hand peeled on the day of analysis. Five plates containing 10 grains were prepared for each sample, corresponding to a total of 50 grains per sample. All plates were incubated at 30 °C for 42 h, and the results were expressed as total percentage grains infected with fungi. A yellow/orange color on the reverse side of the colony was used to indicate presumptive *Aspergillus flavus/parasiticus*.

#### 3.2.5. Fatty Acid Composition

Peanut crude oils were methylated according to the method described by Hartman and Lago [51] and analyzed as described by the Ce 1f-96 method from AOCS [52]. Approximately 100 mg of the crude peanut oil was weighed into a 15 mL screw-top test tube, then 5 mL of internal standard (tridecanoic acid in n-hexane, 2.5 mg/mL) was added, and the contents were shaken. Then 2 mL of methanolic sodium hydroxide solutions (20 mg/mL) was added and the contents were shaken vigorously for 30 s. Then the screw-top test tube was heated in a water bath (70 °C) for five minutes. The content was cooled down under flowing water (30 °C –40 °C) and 2.5 mL of methanolic ammonium chloride solution (33 mg/mL) was added to the tube and the contents were shaken and heated in a water bath (70 °C) for five minutes. The content was cooled down under flowing water (30 °C–40 °C) and the supernatant containing the methyl esters was then decanted and fed to the gas chromatograph. An HP 5890 Series II gas chromatograph (Hewlett-Packard, Palo Alto, CA, USA) equipped with a flame ionization detector (FID) and a split injector was used. Separation was performed in a capillary fused-silica column (100 m × 0.25 mm × 0.2 μm, Agilent J & W GC Columns, Palo Alto, CA, USA) at 130 °C (isothermal). Hydrogen flowed at a rate of 1.5 mL/min as the carrier gas. The injection temperature was 270 °C, and the detector temperature was 280 °C.

#### 3.2.6. Total Phenolic Contents

Total phenolic contents were analyzed in methanolic extracts with a concentration of 0.40 mg/mL, according to the Folin-Ciocalteu method [23], described by Camargo *et al*. [47]. First, 0.50 g of ground peanuts in 50.00 mL of methanol was shaken for 20 min at room temperature. Second, the extract was centrifuged at 700 rpm for 15 min, and the supernatant was collected and transferred to a volumetric flask. Third, the solution was diluted to a volume of 50.00 mL and was used as the final extract. Fourth, the extract (0.50 mL), deionized water (4.0 mL), and Folin-Ciocalteu reagent (0.50 mL) were added into flasks and mixed thoroughly. After 3 min, 1.00 mL of a Na_2_CO_3_ saturated solution (20.00 g in 70.00 mL of water) was added, and the mixture was shaken in a water bath at 37 °C for 30 min. Finally, the absorbance was determined at 760 nm using a Shimadzu UV-1800 spectrophotometer (Shimadzu Corporation; Japan). The results were expressed as milligram gallic acid equivalents.

#### 3.2.7. ABTS Free Radical Scavenging Activity

In this assay from Re *et al*. [24] and described by Camargo *et al*. [47] the ABTS radical (2,2′-azino-bis(3-ethylbenzothiazoline-6-sulphonic acid), which is generated by oxidation with potassium persulfate, was utilized in ethanolic extracts. A 7.00 mM stock solution of ABTS was prepared one day before the analysis, and the working solution was prepared by diluting the stock solution to 0.70 ± 0.02 nm at an absorbance wavelength of 734 nm. The extracts were obtained from 0.75 g ground peanuts and 10.00 mL of ethanol that were shaken for 15 minutes and centrifuged for 10 min at 2000 rpm. The supernatant (20.00 μL) was added into a quartz cuvette along with the ABTS radical solution (2.00 mL), and the absorbance was determined at 734 nm for 6 min using a Shimadzu UV-1800 spectrophotometer (Shimadzu Corporation; Japan). ABTS free radical scavenging activity was calculated using the equation below:

$$\text{ABTS free radical scavenging activity }(\%)=[(Abs_{\text{control}}-Abs_{\text{sample}})/(Abs_{\text{control}})]\times 100$$

where *Abs*_control_ is the absorbance of ABTS radical + ethanol; *Abs*_sample_ is the absorbance of ABTS radical + peanut extract or trolox. The results were expressed as TEAC.

#### 3.2.8. Statistical Analysis

A completely randomized design with three replicates per treatment was used. Analysis of variance and Tukey’s test (*p* < 0.05) were performed with SAS software, and correlation analyses (*p* < 0.01) and (*p* < 0.05) were carried out using the ASSISTAT 7.6 program.

## 4. Conclusions

Darkening was observed in in-shell and blanched samples. The color of in-shell samples was the most affected by storage time. Regarding water activity, in-shell and blanched samples presented different responses to storage time to achieve their equilibrium point. Mycotoxic fungi were detected in non-irradiated blanched samples but not in the gamma-irradiated samples. In the present study 5.2 kGy was a suitable dose to prevent mycotoxic fungi growth. No fungi were detected in the in-shell samples, whether they were irradiated or not. Thus, this feedstock was shown to be the best choice. Negative correlation was found between storage time and *L* *, polyunsaturated fatty acids content, total phenolic contents, and antioxidant activity. No correlation between gamma radiation and these nutraceuticals or even water activity and color was observed. Thus, irradiation protected against MF growth (microbiological safety) and retained both the polyunsaturated fatty acids and polyphenols present in the sample.

## Figures and Tables

**Table 1:** Peanut skin color 1 of gamma-irradiated and stored peanuts.

Color	Peanuts	*T*^2^	0.0 kGy	5.2 kGy	7.2 kGy	10.0 kGy
*L* *	In-shell	0	44.72 ± 1.56 Aab 3,4	43.60 ± 2.82 Ab	41.89 ± 0.58 Ab	48.03 ± 0.40 Aa
		3	41.97 ± 1.44 A	43.75 ± 1.33 A	40.02 ± 2.53 AB	41.85 ± 2.36 B
		6	43.57 ± 1.54 A	42.03 ± 2.98 A	40.36 ± 0.28 AB	40.98 ± 0.42 BC
		9	41.79 ± 1.01 Aa	40.80 ± 0.62 Aab	37.21 ± 1.93 Bc	37.64 ± 1.26 Cbc
		12	26.72 ± 0.23 Ba	24.27 ± 1.04 Bb	26.06 ± 0.54 Cab	25.44 ± 1.19 Dab

	Blanched	0	60.21 ± 3.23 AB	58.21 ± 1.26 A	62.20 ± 0.19 A	58.02 ± 0.72 C
		3	59.14 ±0.46 ABb	60.07 ± 2.40 Ab	53.68 ± 1.07 Bc	64.69 ± 0.52 ABa
		6	57.48 ± 0.67 Bb	58.22 ± 1.40 Ab	60.27 ± 3.57 Ab	66.20 ± 0.65 Aa
		9	62.40 ± 0.24 A	61.17 ± 2.64 A	61.82 ± 0.85 A	62.47 ± 0.49 B
		12	48.61 ± 0.56 C	52.40 ± 1.54 B	48.48 ± 1.50 C	49.25 ± 2.17 D

Chroma	In-shell	0	25.20 ± 1.35 B	25.12 ± 1.25 BC	26.80 ± 2.84 AB	28.60 ± 1.40 A
		3	27.88 ± 1.55 AB	30.44 ± 1.89 A	27.27 ± 0.32 AB	28.04 ± 2.11 AB
		6	29.51 ± 1.89 AB	28.67 ± 2.16 AB	29.99 ± 1.34 A	31.29 ± 1.97 A
		9	30.02 ± 1.65 A	28.33 ± 2.28 AB	27.67 ± 3.53 AB	29.87 ± 1.67 A
		12	25.32 ± 1.90 B	22.40 ± 1.18 C	23.76 ± 1.47 B	23.03 ± 1.13 B

	Blanched	0	20.38 ± 1.29 A	19.91 ± 2.11	20.90 ± 0.94 A	19.74 ± 0.26 DC
		3	21.30 ± 0.43 Aab	20.97 ± 1.43 b	20.23 ± 0.16 Ab	22.98 ± 0.25 ABa
		6	19.41 ± 0.51 ABb	20.11 ± 1.48 b	21.17 ± 0.98 Aab	24.09 ± 2.04 Aa
		9	20.92 ± 0.55 A	21.13 ± 0.76	20.72 ± 0.42 A	20.91 ± 1.25 BC
		12	15.57 ± 3.20 B	21.01 ± 5.51	17.09 ± 0.78 B	16.77 ± 1.23 D

°Hue	In-shell	0	53.05 ± 2.13	55.67 ± 4.48	52.74 ± 2.63	57.22 ± 2.55
		3	56.21 ± 2.42 ab	51.82 ± 1.58 b	56.48 ± 0.94 ab	56.96 ± 1.89 a
		6	56.51 ± 2.81	57.19 ± 1.71	55.51 ± 0.64	56.37 ± 3.87
		9	55.32 ± 0.66	57.17 ± 1.09	53.58 ± 2.93	57.32 ± 1.88
		12	57.79 ± 1.34 a	56.02 ± 0.74 ab	54.03 ± 0.98 b	53.82 ± 0.22 b

	Blanched	0	86.46 ± 1.23 ab	85.46 ± 0.79 b	88.22 ± 0.50 a	86.23 ± 0.81 Aab
		3	84.67 ± 0.66	86.58 ± 1.04	85.14 ± 3.29	85.52 ± 0.50 AB
		6	85.97 ± 0.94	85.76 ± 0.76	85.14 ± 0.82	86.29 ± 0.53 A
		9	85.54 ± 0.25	85.69 ± 0.79	85.01 ± 1.05	85.93 ± 0.44 A
		12	86.76 ± 0.80 a	85.09 ± 1.15 ab	84.28 ± 0.58 b	84.28 ± 0.58 Bb

1 Data represent the means of triplicate analysis for each sample ± standard deviations;

2 *T* is storage time in months;

3 means with the same capital letters within a column are not significantly different according to Tukey’s multiple test (*p* < 0.05);

4 means with the same small letter within a row are not significantly different according to Tukey’s multiple test (*p* < 0.05).

**Table 2:** Pearson’s correlation between gamma radiation dose and color parameters and between storage time and color parameters

	Peanut	*L* *	Chroma	°Hue
Irradiation × Color	In-shell	−0.3308 ^ns^	0.0862 ^ns^	−0.0499 ^ns^
	Blanched	0.2435 ^ns^	0.2383 ^ns^	−0.0286 ^ns^
Storage time × Color	In-shell	−0.8147 **	−0.2545 ^ns^	0.1596 ^ns^
	Blanched	−0.4401 *	−0.3894 ^ns^	−0.2877 ^ns^

** significant (*p* < 0.01);

* significant (*p* < 0.05);

ns: non-significant.

**Table 3:** Water activity 1 of gamma-irradiated and stored peanuts.

Peanuts	*T* 2	0.0 kGy	5.2 kGy	7.2 kGy	10.0 kGy
In-shell	0	0.673 ± 0.011 A 3,4	0.682 ± 0.013 A	0.674 ± 0.003 A	0.687 ± 0.004 A
	3	0.584 ± 0.011 Cb	0.586 ± 0.004 Cab	0.605 ± 0.005 Ca	0.570 ± 0.016 Cb
	6	0.649 ± 0.005 B	0.642 ± 0.004 B	0.644 ± 0.001 B	0.649 ± 0.002 B
	9	0.584 ± 0.000 Ca	0.587 ± 0.006 Ca	0.578 ± 0.000 Da	0.563 ± 0.003 Cb
	12	0.542 ± 0.008 Dab	0.523 ± 0.013 Dbc	0.553 ± 0.002 Ea	0.519 ± 0.011 Dc

Blanched	0	0.425 ± 0.001 Dc	0.455 ± 0.004 Ea	0.437 ± 0.001 Eb	0.437 ± 0.005 Eb
	3	0.524 ± 0.008 Ca	0.500 ± 0.002 Db	0.493 ± 0.001 Db	0.504 ± 0.002 Db
	6	0.627 ± 0.003 Ab	0.617 ± 0.004 Ac	0.642 ± 0.003 Aa	0.591 ± 0.003 Bd
	9	0.622 ± 0.002 Aa	0.588 ± 0.001 Bc	0.602 ± 0.001 Bb	0.616 ± 0.004 Aa
	12	0.554 ± 0.001 B	0.559 ± 0.004 C	0.562 ± 0.022 C	0.571 ± 0.018 C

1 Data represent the means of triplicate analysis for each sample ± standard deviations;

2 *T* is storage time in months;

3 means with the same capital letters within a column are not significantly different according to Tukey’s multiple test (*p* < 0.05);

4 means with the same small letter within a row are not significantly different according to Tukey’s multiple test (*p* < 0.05).

**Table 4:** Fungal infection (%) 1 in gamma-irradiated and stored peanuts.

		Positive samples (%)			

Peanuts	*T*^2^	0.0 kGy	5.2 kGy	7.2 kGy	10.0 kGy
In-shell	0	0	0	0	0
	3	0	0	0	0
	6	0	0	0	0
	9	0	0	0	0
	12	0	0	0	0

Blanched	0	2	0	0	0
	3	8	0	0	0
	6	0	0	0	0
	9	2	0	0	0
	12	0	0	0	0

1 Each data point represents the results of 5 samples of 10 kernels;

2 *T* is storage time in months.

**Table 5:** Fatty acid compositions (g/100 g) 1 of gamma-irradiated and stored peanuts.

Fatty acids	Peanuts	*T* 2	0.0 kGy	5.2 kGy	7.2 kGy	10.0 kGy
C16:0	In-shell	0	13.33 ± 0.02 3,4	12.65 ± 0.70	12.33 ± 0.38	13.62 ± 0.79 A
		3	12.87 ± 0.26	12.33 ± 0.50	11.89 ± 0.66	13.29 ± 0.81 A
		6	12.88 ± 0.63	12.88 ± 0.23	12.62 ± 0.41	12.41 ± 0.57 AB
		9	11.75 ± 0.38 b	11.83 ± 0.37 ab	12.56 ± 0.09 a	11.59 ± 0.19 Bb
		12	13.36 ± 1.58	11.95 ± 0.79	12.16 ± 1.08	13.00 ± 0.08 AB

	Blanched	0	12.14 ± 0.54	12.78 ± 1.31	11.79 ± 0.98	12.57 ± 0.03 ABC
		3	11.65 ± 0.05 b	12.33 ± 0.22 ab	12.44 ± 0.56 ab	13.18 ± 0.48 Aa
		6	11.68 ± 0.17	12.06 ± 0.59	12.53 ± 0.80	12.23 ± 0.20 BC
		9	12.62 ± 0.27	12.53 ± 2.34	11.81 ± 0.43	12.01 ± 0.47 C
		12	12.28 ± 0.53 ab	11.92 ± 0.08 b	12.04 ± 0.22 b	12.98 ± 0.02 ABa

C18:0	In-shell	0	1.47 ± 0.03 b	1.64 ± 0.02 Ba	1.64 ± 0.02 Ba	1.57 ± 0.08 Bab
		3	1.59 ± 0.02 b	1.58 ± 0.03 BCb	1.55 ± 0.02 Db	1.65 ± 0.02 ABa
		6	1.53 ± 0.02	1.55 ± 0.01 C	1.58 ± 0.01 CD	1.57 ± 0.06 AB
		9	1.46 ± 0.10 b	1.61 ± 0.01 BCa	1.59 ± 0.01 Cab	1.57 ± 0.02 Bab
		12	1.63 ± 0.16	1.73 ± 0.02 A	1.75 ± 0.02 A	1.75 ± 0.11 A

	Blanched	0	2.08 ± 0.00 ABb	2.36 ± 0.18 Aa	2.14 ± 0.08 Aab	2.21 ± 0.04 Aa
		3	1.99 ± 0.06 B	1.97 ± 0.02 BC	1.90 ± 0.00 BC	1.94 ± 0.03 B
		6	1.83 ± 0.04 C	1.87 ± 0.02 C	1.84 ± 0.07 C	1.88 ± 0.05 B
		9	2.13 ± 0.03 A	2.08 ± 0.13 BC	2.07 ± 0.12 AB	2.21 ± 0.00 A
		12	2.14 ± 0.00 A	2.18 ± 0.02 AB	2.13 ± 0.03 A	2.14 ± 0.04 A

C18:1	In-shell	0	44.94 ± 0.26 C	44.93 ± 0.25 C	45.22 ± 0.54 D	44.42 ± 0.10 C
		3	47.06 ± 0.26 Ba	46.95 ± 0.09 BCa	45.95 ± 0.46 CDb	47.04 ± 0.02 Ba
		6	46.86 ± 0.13 B	46.61 ± 0.18 BC	46.99 ± 0.07 BC	46.92 ± 0.30 B
		9	49.71 ± 0.62 Aab	51.32 ± 1.75 Aa	50.35 ± 1.03 Aab	48.03 ± 0.42 Ab
		12	46.90 ± 0.42 Bb	48.04 ± 0.38 Ba	47.99 ± 0.03 Ba	48.13 ± 0.13 Aa

	Blanched	0	44.92 ± 0.23 Bab	43.66 ± 0.78 Bb	46.13 ± 0.47 Da	44.06 ± 0.56 Db
		3	48.86 ± 0.23 A	48.76 ± 0.25 A	47.57 ± 1.30 CD	48.38 ± 0.23 C
		6	48.27 ± 0.16 A	48.53 ± 0.12 A	48.36 ± 0.05 BC	48.50 ± 0.39 C
		9	44.64 ± 1.36 Bb	47.62 ± 3.48 ABab	50.36 ± 0.67 Aa	50.92 ± 0.45 Aa
		12	49.56 ± 0.27 A	49.69 ± 0.31 A	49.37 ± 0.28 AB	49.80 ± 0.37 B

C18:2	In-shell	0	37.10 ± 0.32 A	37.74 ± 0.53 A	37.70 ± 0.57 A	37.66 ± 0.44 A
		3	35.06 ± 0.15 Bab	35.84 ± 0.53 ABab	36.60 ± 1.27 ABa	34.62 ± 0.39 BCb
		6	35.59 ± 0.46 B	35.48 ± 0.11 AB	35.30 ± 0.21 BC	35.37 ± 0.01 B
		9	34.79 ± 0.08 BCa	31.06 ± 2.04 Cb	31.91 ± 0.87 Dab	34.80 ± 0.40 BCa
		12	34.16 ± 0.36 C	33.57 ± 0.25 BC	33.77 ± 0.35 CD	33.40 ± 0.96 C

	Blanched	0	37.63 ± 0.22 A	37.35 ± 0.74 A	35.86 ± 1.19 A	37.53 ± 0.32 A
		3	33.66 ± 0.78 BC	32.98 ± 0.14 BC	34.53 ± 1.92 AB	33.02 ± 0.10 C
		6	34.46 ± 0.53 Ba	33.98 ± 0.05 BCab	33.44 ± 0.36 BCb	33.53 ± 0.01 Bb
		9	37.42 ± 1.78 Aa	34.66 ± 2.25 ABab	32.16 ± 0.76 BCb	30.85 ± 0.11 Cb
		12	31.31 ± 0.63 C	31.47 ± 0.39 C	31.42 ± 0.03 C	30.50 ± 0.25 C

C20:0	In-shell	0	0.52 ± 0.03	0.54 ± 0.04 C	0.56 ± 0.02 C	0.50 ± 0.04 B
		3	0.65 ± 0.02	0.63 ± 0.00 BC	0.69 ± 0.01 AB	0.65 ± 0.07 AB
		6	0.72 ± 0.10	0.63 ± 0.00 BC	0.62 ± 0.03 BC	0.66 ± 0.05 AB
		9	0.48 ± 0.06 b	0.69 ± 0.02 ABab	0.65 ± 0.03 ABCab	0.83 ± 0.17 Aa
		12	0.68 ± 0.19	0.79 ± 0.08 A	0.74 ± 0.08 A	0.69 ± 0.12 AB

	Blanched	0	0.61 ± 0.05 C	0.60 ± 0.09 AB	0.70 ± 0.04 B	0.62 ± 0.01 D
		3	0.81 ± 0.07 AB	0.81 ± 0.04 AB	0.73 ± 0.03 B	0.73 ± 0.02 C
		6	0.76 ± 0.04 ABC	0.75 ± 0.08 AB	0.74 ± 0.11 B	0.78 ± 0.02 B
		9	0.62 ± 0.12 BC	0.65 ± 0.17 B	0.72 ± 0.06 B	0.75 ± 0.02 BC
		12	0.89 ± 0.02 A	0.92 ± 0.02 A	0.93 ± 0.01 A	0.90 ± 0.00 A

C22:0	In-shell	0	0.77 ± 0.03	0.80 ± 0.04 C	0.83 ± 0.01 B	0.73 ± 0.06 B
		3	0.89 ± 0.01	0.87 ± 0.02 BC	0.95 ± 0.02 AB	0.90 ± 0.08 AB
		6	1.04 ± 0.14	0.90 ± 0.00 BC	0.91 ± 0.04 AB	0.95 ± 0.07 A
		9	0.78 ± 0.00 c	0.98 ± 0.01 ABa	0.91 ± 0.04 ABb	0.94 ± 0.02 ABab
		12	0.95 ± 0.25	1.03 ± 0.09 A	1.02 ± 0.13 A	0.92 ± 0.13 AB

	Blanched	0	0.73 ± 0.05 B	0.73 ± 0.08	0.81± 0.03 B	0.75 ± 0.01 C
		3	0.89 ± 0.07 A	0.89 ± 0.03	0.82 ± 0.03 B	0.82 ± 0.01 BC
		6	0.84 ± 0.04 AB	0.88 ± 0.11	0.84 ± 0.07 AB	0.85 ± 0.02 AB
		9	0.74 ± 0.12 B	0.79 ± 0.20	0.90 ± 0.06 AB	0.90 ± 0.01 A
		12	0.95 ± 0.05 A	0.98 ± 0.02	0.94 ± 0.01 A	0.92 ± 0.05 A

C22:1	In-shell	0	1.45 ± 0.46	1.21 ± 0.21 C	1.23 ± 0.19	1.14 ± 0.01 B
		3	1.35 ± 0.04 ab	1.26 ± 0.10 BCb	1.61 ± 0.06 a	1.31 ± 0.19 ABb
		6	1.20 ± 0.02 b	1.35 ± 0.04 BCab	1.36 ± 0.15 ab	1.45 ± 0.06 ABa
		9	0.81 ± 0.01 b	1.69 ± 0.02 ABa	1.46 ± 0.14 a	1.53 ± 0.25 Aa
		12	1.57 ± 0.60	1.93 ± 0.30 A	1.73 ± 0.30	1.07 ± 0.03 B

	Blanched	0	1.32 ± 0.33 AB	1.84 ± 0.89	1.81 ± 0.28 AB	1.58 ± 0.31 AB
		3	1.52 ± 0.21 AB	1.57 ± 0.12	1.37 ± 0.05 B	1.33 ± 0.09 B
		6	1.47 ± 0.06 AB	1.36 ± 0.29	1.51 ± 0.21 B	1.50 ± 0.06 AB
		9	1.24 ± 0.28 B	1.22 ± 0.38	1.44 ± 0.27 B	1.64 ± 0.08 AB
		12	1.91 ± 0.07 Ab	1.90 ± 0.06 b	2.06 ± 0.04 Aa	1.82 ± 0.01 Ab

C24:0	In-shell	0	0.43 ± 0.11 AB	0.50 ± 0.10 C	0.49 ± 0.11 C	0.37 ± 0.06
		3	0.54 ± 0.05 ABb	0.54 ± 0.07 Cb	0.76 ± 0.04 ABa	0.55 ± 0.07 b
		6	0.48 ± 0.01 ABb	0.60 ± 0.04 BCa	0.62 ± 0.07 ABCa	0.66 ± 0.02 a
		9	0.23 ± 0.01 Bb	0.81 ± 0.03 ABa	0.56 ± 0.03 BCa	0.72 ± 0.20 a
		12	0.74 ± 0.32 A	0.96 ± 0.17 A	0.84 ± 0.17 A	0.65 ± 0.19

	Blanched	0	0.56 ± 0.13 Bb	0.68 ± 0.10 ABab	0.75 ± 0.03 Ba	0.68 ± 0.03 BCab
		3	0.64 ± 0.09 B	0.69 ± 0.12 AB	0.63 ± 0.02 BC	0.60 ± 0.06 C
		6	0.69 ± 0.01 B	0.56 ± 0.17 AB	0.73 ± 0.11 B	0.72 ± 0.05 B
		9	0.59 ± 0.14 B	0.46 ± 0.22 B	0.53 ± 0.01 C	0.66 ± 0.02 B
		12	0.96 ± 0.02 Ab	0.95 ± 0.08 Ab	1.11 ± 0.00 Aa	0.93 ± 0.01 Ab

SFA	In-shell	0	16.52 ± 0.12 A	16.12 ± 0.49	15.85 ± 0.21	16.78 ± 0.55 AB
		3	16.54 ± 0.36 A	15.95 ± 0.52	15.83 ± 0.75	17.03 ± 0.56 A
		6	16.65 ± 0.90 A	16.57 ± 0.25	16.35 ± 0.29	16.25 ± 0.37 AB
		9	14.69 ± 0.56 Bb	15.92 ± 0.30 a	16.28 ± 0.03 a	15.65 ± 0.56 Bab
		12	17.37 ± 0.67 A	16.45 ± 0.43	16.51 ± 0.68	17.01 ± 0.46 AB

	Blanched	0	16.12 ± 0.32 BC	17.15 ± 0.85	16.19 ± 1.00	16.83 ± 0.06 B
		3	15.96 ± 0.34 BCb	16.69 ± 0.01 ab	16.52 ± 0.49 ab	17.26 ± 0.42 ABa
		6	15.80 ± 0.30 C	16.13 ± 0.23	16.73 ± 0.50	16.47 ± 0.34 B
		9	16.70 ± 0.15 AB	16.51 ± 1.62	16.04 ± 0.19	16.59 ± 0.48 B
		12	17.22 ± 0.44 Ab	16.95 ± 0.02 b	17.15 ± 0.21 b	17.88 ± 0.11 Aa

PUFA	In-shell	0	37.10 ± 0.32 A	37.74 ± 0.53 A	37.70 ± 0.57 A	37.66 ± 0.44 A
		3	35.06 ± 0.15 Bab	35.84 ± 0.53 ABab	36.60 ± 1.27 ABa	34.62 ± 0.39 BCb
		6	35.59 ± 0.46 B	35.48 ± 0.11 AB	35.30 ± 0.21 BC	35.37 ± 0.01 B
		9	34.79 ± 0.08 BCa	31.06 ± 2.04 Cb	31.91 ± 0.87 Dab	34.80 ± 0.40 BCa
		12	34.16 ± 0.36 C	33.57 ± 0.25 BC	33.77 ± 0.35 CD	33.40 ± 0.96 C

	Blanched	0	37.63 ± 0.22 A	37.35 ± 0.74 A	35.86 ± 1.19 A	37.53 ± 0.32 A
		3	33.66 ± 0.78 BC	32.98 ± 0.14 BC	34.53 ± 1.92 AB	33.02 ± 0.10 C
		6	34.46 ± 0.53 Ba	33.98 ± 0.05 BCab	33.44 ± 0.36 BCb	33.53 ± 0.01 Bb
		9	37.42 ± 1.78 Aa	34.66 ± 2.25 ABab	32.16 ± 0.76 BCb	30.85 ± 0.11 Cb
		12	31.31 ± 0.63 C	31.47 ± 0.39 C	31.42 ± 0.03 C	30.50 ± 0.25 C

MUFA	In-shell	0	46.38 ± 0.20 Ca	46.14 ± 0.04 Ca	46.45 ± 0.35 Da	45.56 ± 0.11 Cb
		3	48.40 ± 0.21 Ba	48.22 ± 0.01 BCab	47.57 ± 0.52 CDb	48.35 ± 0.18 Ba
		6	48.06 ± 0.15 B	47.96 ± 0.14 BC	48.35 ± 0.08 BC	48.37 ± 0.36 B
		9	50.52 ± 0.64 Aab	53.01 ± 1.73 Aa	51.81 ± 0.89 Aa	49.56 ± 0.17 Ab
		12	48.47 ± 1.02 B	49.97 ± 0.68 B	49.72 ± 0.34 B	49.20 ± 0.10 A

	Blanched	0	46.24 ± 0.10 Bb	45.50 ± 0.11 Bc	47.94 ± 0.18 Da	45.64 ± 0.26 Dc
		3	50.38 ± 0.44 Aa	50.33 ± 0.13 Aa	48.95 ± 0.87 CDb	49.71 ± 0.32 Cab
		6	49.74 ± 0.22 A	49.89 ± 0.17 AB	49.87 ± 0.24 BC	50.00 ± 0.33 C
		9	45.88 ± 1.64 Bb	48.84 ± 3.86 ABab	51.80 ± 0.95 Aa	52.57 ± 0.36 Aa
		12	51.47 ± 0.19 A	51.59 ± 0.37 A	51.43 ± 0.24 AB	51.62 ± 0.36 B

1 Data represent the means of triplicate analysis for each sample ± standard deviations;

2 *T* is storage time in months. SFA is saturated fatty acids. PUFA is polyunsaturated fatty acids. MUFA is monounsaturated fatty acids;

3 means with the same capital letters within a column are not significantly different according to Tukey’s multiple test (*p* < 0.05);

4 means with the same small letter within a row are not significantly different according to Tukey’s multiple test (*p* < 0.05).

**Table 6:** Pearson’s correlation between the variables: irradiation and fatty acid content or storage time and fatty acid content.

Fatty acids	Irradiation		Storage time	

	In-shell	Blanched	In-shell	Blanched
C16:0	−0.1087 ^ns^	0.2787 ^ns^	−0.3306 ^ns^	−0.0523 ^ns^
C18:0	0.4935 *	0.1000 ^ns^	0.4222 ^ns^	0.0744 ^ns^
C18:1	−0.0611 ^ns^	0.2263 ^ns^	0.7239 **	0.6181 **
C18:2	−0.0561 ^ns^	−0.3834 ^ns^	−0.7870 **	−0.7267 **
C20:0	0.2172 ^ns^	0.2046 ^ns^	0.5927 **	0.6167 **
C22:0	0.0763 ^ns^	0.1771 ^ns^	0.6304 **	0.6505 **
C22:1	0.1355 ^ns^	0.2234 ^ns^	0.3269 ^ns^	0.2738 ^ns^
C24:0	0.2316 ^ns^	0.2085 ^ns^	0.5760 **	0.3933 ^ns^
SFA ***	0.0731 ^ns^	0.4024 ^ns^	0.0731 ^ns^	0.4024 ^ns^
PUFA	−0.0561 ^ns^	−0.3834 ^ns^	−0.7870 **	−0.7267 **
MUFA	−0.0291 ^ns^	0.2385 ^ns^	0.7452 **	0.6397 **

** significant (*p* < 0.01);

* significant (*p* < 0.05);

ns: non significant;

*** SFA is saturated fatty acids; PUFA is polyunsaturated fatty acids, MUFA is monounsaturated fatty acids.

**Table 7:** Total phenolic contents (mg gallic acid/g of dry sample) 1 of irradiated and stored peanuts.

Peanuts	*T* 2	0.0 kGy	5.2 kGy	7.2 kGy	10.0 kGy
In-shell	0	4.62 ± 0.18 Aab 3,4	4.40 ± 0.14 Ab	4.78 ± 0.20 Aab	4.85 ± 0.09 Aa
	3	4.33 ± 0.12 A	4.30 ± 0.13 A	4.27 ± 0.08 B	4.33 ± 0.08 B
	6	3.26 ± 0.05 B	3.22 ± 0.07 B	3.29 ± 0.10 C	3.25 ± 0.10 C
	9	3.24 ± 0.25 B	2.96 ± 0.04 C	3.09 ± 0.12 C	3.27 ± 0.25 C
	12	3.32 ± 0.09 Ba	2.84 ± 0.06 Cc	3.14 ± 0.14 Cab	3.04 ± 0.02 Cbc

Blanched	0	2.52 ± 0.03 A	2.58 ± 0.08 A	2.53 ± 0.04 B	2.68 ± 0.08 B
	3	2.54 ± 0.17 Ab	2.16 ± 0.13 Ac	3.17 ± 0.10 Aa	3.23 ± 0.10 Aa
	6	2.32 ± 0.15 Aa	2.46 ± 0.01 Aa	2.30 ± 0.06 Ca	1.80 ± 0.03 Cb
	9	1.53 ± 0.40 B	1.47 ± 0.44 B	1.61 ± 0.50 C	1.39 ± 0.34 C
	12	1.43 ± 0.03 Ba	1.24 ± 0.00 Bb	1.10 ± 0.01 Cc	1.41 ± 0.04 Ca

1 Data represent the means of triplicate analysis for each sample ± standard deviations;

2 *T* is storage time in months;

3 means with the same capital letters within a column are not significantly different according to Tukey’s multiple test (*p* < 0.05);

4 means with the same small letter within a row are not significantly different according to Tukey’s multiple test (*p* < 0.05).

**Table 8:** 2,2′-Azino-bis(3-ethylbenzothiazoline-6-sulphonic acid (ABTS) free radical scavenging activity (μmol TEAC/g of dry sample) 1 of gamma-irradiated and stored peanuts.

Peanuts	*T* 2	0.0 kGy	5.2 kGy	7.2 kGy	10.0 kGy
In-shell	0	16.97 ± 0.10 A 3,4	16.83 ± 0.08 A	16.80 ± 0.08 A	16.70 ± 0.16 A
	3	14.94 ± 0.32 B	15.21 ± 0.16 B	15.24 ± 0.27 B	15.37 ± 0.39 AB
	6	13.50 ± 0.50 Ca	9.59 ± 0.08 Eb	10.29 ± 0.70 Cb	12.38 ± 1.11 Da
	9	14.17 ± 0.31 BCab	13.50 ± 0.10 Cb	13.78 ± 0.14 Aab	14.65 ± 0.60 BCa
	12	13.79 ± 0.30 Ca	13.17 ± 0.12 Db	13.55 ± 0.28 Aab	13.27 ± 0.04 CDab

Blanched	0	10.36 ± 0.11 b	10.74 ± 0.12 a	10.91 ± 0.12 a	11.02 ± 0.10 a
	3	nd 5	nd	nd	nd
	6	nd	nd	nd	nd
	9	nd	nd	nd	nd
	12	nd	nd	nd	nd

1 Data represent the means of triplicate analysis for each sample ± standard deviations;

2 *T* is storage time in months;

3 means with the same capital letters within a column are not significantly different according to Tukey’s multiple test (*p* < 0.05);

4 means with the same small letter within a row are not significantly different according to Tukey’s multiple test (*p* < 0.05);

5 nd means non detected.
